# Freeze drying microencapsulation using whey protein, maltodextrin and corn powder improved survivability of probiotics during storage

**DOI:** 10.1007/s10068-024-01706-w

**Published:** 2024-09-12

**Authors:** Benjamaporn Poopan, Anongnard Kasorn, Narathip Puttarat, Kittiwut Kasemwong, Ulisa Pachekrepapol, Malai Taweechotipatr

**Affiliations:** 1 Molecular Biology Program, Faculty of Medicine, Srinakharinwirot University, 114 Sukhumvit 23, Wattana District, Bangkok, 10110 Thailand; 2 Department of Basic Medical Science, Faculty of Medicine Vajira Hospital, Navamindradhiraj University, 681 Samsen Road, Wachira Phayaban, Dusit District, Bangkok, 10300 Thailand; 3 Center of Excellence in Probiotics, Srinakharinwirot University, 114 Sukhumvit 23, Wattana District, Bangkok, 10110 Thailand; 4 NANOTEC Research Unit, National Nanotechnology Center, National Science and Technology Development Agency, 130 Thailand Science Park, Paholyothin Road, Khlong Luang, Pathumthani, 12120 Thailand; 5 Division of Food Science and Nutrition, Faculty of Agricultural Product Innovation and Technology, Srinakharinwirot University, 63 Village No.7, Khlong 16 Road, Ongkharak, Nakornnayok, 26120 Thailand; 6 Department of Microbiology, Faculty of Medicine, Srinakharinwirot University, 114 Sukhumvit 23, Wattana District, Bangkok, 10110 Thailand

**Keywords:** Microencapsulation, Freeze drying, Probiotics, Corn powder, Mallard reaction

## Abstract

Various studies demonstrated that probiotics play important roles in maintaining the balance of microorganisms in the body. Some strains produce bile salt hydrolase enzyme (BSH), which is an indirect mechanism for lowering cholesterol. BSH-producing probiotics as a supplement might be an alternative way to help reducing cholesterol in the body. The aim of this study was to investigate the effects of different microcapsule formulations with selected vegetable powders on growth characteristics of 3 Thai probiotic strains, *Lactobacillus gasseri* TM1, *Lacticaseibacillus rhamnosus* TM7, and *L. rhamnosus* TM14. Probiotics were cultured in MRS broth supplemented with 5 vegetable powders. Corn powder significantly increased growth rate of probiotics from 10^9^ to 10^12^ CFU/ml. Therefore, different microcapsule formulations by Maillard reaction of whey protein isolate and maltodextrin mixed with and without corn powder were studied. The results showed that probiotic microcapsules formulated with corn powder significantly effectively sustained probiotic viability under gastrointestinal and storage conditions.

**Supplementary Information:**

The online version contains supplementary material available at 10.1007/s10068-024-01706-w.

## Introduction

Probiotics are beneficial commensal microbes in human. Microbiota is a large community of microorganisms that grows in the stomach, small and large intestines. The majority of these microorganisms which are beneficial to human body are members of probiotics. The definition of probiotics defined by the Food and Agriculture Organization of the United Nations/World Health Organization (FAO/WHO) with minor modified by the International Scientific Association for Probiotics and Prebiotics (ISAPP) is “live microorganisms that when administered in adequate amounts confer a health benefit on the host” (Salminen et al., 2007).

*Lactobacillus* spp*.*, *Bifidobacterium* spp*.*, and *Enterococcus* spp. are the most common probiotics*.* These probiotics produce lactic acid via anaerobic respiration process. Probiotics are found in dairy-based foods such as yogurt and cheese, pickled vegetables, bread, fermented food, and sausages. According to the principle of Generally Recognized As Safe (GRAS), dietary probiotics with health benefits must have certain properties such as able to colonize the gastrointestinal (GI) tract, improve intestinal function, produce nutrients, protect host from pathogens, and their toxins and modulate immune system (De Angelis et al., 2019).

Microencapsulation is an effective technology to help protect probiotics from stress conditions (Ammara et al., 2014). During manufacturing, storage, and passage through GI tract, probiotic cells are wrapped in a physical barrier to protect organisms from external conditions or the environment. The most commonly used substances in the microencapsulation process are maltodextrin (MD). MD is carbohydrate that forms strong covalent bonds and possess prebiotic characteristics served as the wall of the microcapsule through the Maillard reaction. Recent research has been incorporated whey protein isolate (WPI) in microencapsulation process. WPI, which is easily combined with carbohydrates and form a strong bond to the microcapsule walls, not toxic to the body and is digested thoroughly in the stomach, leading to a good release of bacteria to target organs (Liu et al., 2017). Moreover, WPI is also a strong source of functional proteins with good physicochemical characteristics which contributes to formation of a structure that links the molecule with other biopolymers (Zhang et al., 2020). However, MD or WPI encapsulation alone may not sustain acidic environments and may not tolerate conditions in the manufacturing process. More than two components are typically blended during the encapsulation process to produce a more stable and stronger capsule structure.

Corn which is abundant in Northern Thailand is a vegetable enriched with nutrients especially soluble corn fiber (SCF). SCF, a non-digestible oligosaccharide in digestive tract, is considered as prebiotics. Naturally, probiotics living in the GI tract digest this fiber into small molecules which can be absorbed into the intestine. To be classified as prebiotics, nutrients need to meet three criteria as followed. They must be digested by probiotics and are benefit to the body. They must not be digested or absorbed in the stomach and small intestine. The common prebiotics include oligosaccharide, raffinose, inulin and non-starch polysaccharides (Floch, 2018). The oligosaccharide can be synthesized by enzymes catalyst of hydrolysis and trans-glycosylation of carbohydrate-modifying enzymes. (Perugino et al., 2004).

A previous study from our group reported cholesterol lowering property of *L. gasseri* TM1*, L. rhamnosus* TM7, and *L. rhamnosus* TM14 isolated from milk supplied by Thailand dairy farm cooperative (on submission). These strains of probiotics produce bile salt hydrolase (BSH) enzyme which is a precursor of bile salt production in the body. BSH activity in probiotics is correlated with the ability to lower serum cholesterol levels in hypercholesterolemic patients (Hernández-Gómez et al., 2021). According to their property, these strains of probiotics were used in this study. In addition, this study also investigated the effects of different vegetable powders (beetroot, bok choy, pumpkin, corn and tomato) on viability of probiotics in order to select a suitable vegetable powder to incorporate into probiotic microcapsules. Stability of microencapsulated probiotics during product storage and the survival of probiotics in microcapsules under simulated GI conditions also examined.

## Materials and methods

### Characterization of probiotics

Three strains of probiotics with the ability to produce bile-salt hydrolase enzyme were selected for this study. *Lactobacillus gasseri* TM1*, Lacticaseibacillus rhamnosus* TM7 and *L. rhamnosus* TM14 were obtained from Center of Excellence in Probiotics, Srinakharinwirot University, Thailand. These probiotics were stored in De Man, Rogosa, and Sharpe (MRS) broth (Himedia, India) supplemented with 30% (v/v) glycerol at – 80 °C.

### Effect of vegetable powders on probiotic growth

Probiotics (10^8^ CFU/mL) were added to MRS broth culture medium or MRS broth with 5% w/v vegetable powders (beetroot, bok choy, pumpkin, corn and tomato) supplied by Chiangmai Bioveggie Co., Ltd., Thailand. Probiotics growths were measured by the absorbance at 600 nm of the probiotic solution every 2 h at 37 °C for 48 h and reported as a growth curve. A vegetable powder that efficiently enhanced the growth of probiotics was selected for subsequent studies.

### Formulation of probiotic microcapsules

Selection of wall materials in microcapsules is important for probiotics encapsulation to stabilize viability of probiotics during difference conditions (Kambhampati et al., 2023). The mixture of whey protein isolate (WPI) and maltodextrin (MD) are more suitable for microcapsule production than MD alone. WPI solution was prepared in sterile distilled water and heated at 65 °C for 30 min. Then, MD was added and followed by magnetic stirring and heating at 90°C to induce Maillard reaction for 2–4 h. The Maillard reaction, which occurs during the heating process of this mixture, improves the thermal stability of WPI (Liu et al., 2017). Then, the temperature of the mixture was adjusted to 25 °C. Corn powder was then added and stirred for 15 min. After that, probiotics were added into the mixture, stirred for 5 min to form encapsulation, and stored at – 80 °C overnight. The mixture was freeze-dried (Cool Safe Scanvac, LaboGene, Allerod, Denmark) for 74 h at a pressure < 0.4 mbar using a temperature ramp starting from 42 °C up to 20 °C optimized according to the T_g_ and T’_g_ of the initial aqueous systems.

In this study, viabilities of probiotics in microcapsules from different materials were compared before and after the microencapsulation process by freeze drying. Three formulas of microencapsulation were compared in this study including maltodextrin alone (MD), the mixture of MD-WPI (MW), and the mixture of MD-WPI and corm powder (MWC). In addition, free cells were used as a control.

### Probiotic microencapsulated yields

The encapsulated probiotic cells and probiotic cell suspensions were used in this experiment in order to measure probiotic cells viability before and after freeze drying. The encapsulated probiotic cells were released with PBS, pH 7.2, and then all samples were 10 -fold serially diluted with PBS, pH 7.2 and spread on MRS agar with CaCO_3_ followed by anaerobic incubation at 37 °C for 48 h (Lulwah et al., 2016).

### Morphological analysis using scanning electron microscope

The morphology of microcapsules was analyzed using scanning electron microscope (Hitachi High -Technologies, Japan). Microcapsule samples were prepared on stub using carbon tape and coated with gold particle for electrical conductivity. Condition of electron in vacuum was set at electric potential acceleration of 20 kV.

### Survival of probiotics in microcapsules in simulated gastrointestinal conditions

The simulated gastric juice and simulated intestinal juice were used as a gastrointestinal condition model to study the survival of probiotics in microcapsule products after exposing to the gastrointestinal condition. Initially, the artificial gastric juice was prepared using 0.2% (w/v) sodium chloride (NaCl) solution consisting of 0.35% (w/v) pepsin (HiMedia, India), and the pH was adjusted with 1M of hydrochloric acid (HCl) to pH 2.0. The artificial intestinal juice was prepared by using 0.2% (w/v) NaCl solution consisting of 0.1% (w/v) trypsin (HiMedia, India), 1.0% (w/v) oxgall and 1.1% w/v sodium bicarbonate (NaH_2_CO_3_), and the pH was adjusted with 1 M of sodium hydroxide (NaOH) to pH 8.0. One gram of encapsulated probiotic cells and probiotic cell suspensions were added to artificial gastric juice, followed by incubation at 37 °C for 2 h (Chun et al., 2014).

### Survivability of encapsulated probiotic cells during storage

To study the survivability of microencapsulated probiotics during storage, the encapsulated probiotic cells suspensions were stored at 4 °C and 25 °C for 1 years. Every month during storage, 1 g of encapsulated probiotic cells suspensions were evaluated for probiotic viability. Briefly, the encapsulated probiotic cells were counted by suspending 1 g of sample in 9 mL of PBS, pH 7.2. Then, the suspensions were tenfold serially diluted and spread on MRS agar with CaCO_3_ followed by an anaerobic incubation at 37 °C for 48 h. The numbers of colonies were counted and reported as CFU/g of viable probiotic cells each week (Nagyzbekkyzy et al., 2016).

### Bile salt hydrolase enzyme activity of probiotics under each experimental condition

One gram of encapsulated probiotic cells suspensions was evaluated for probiotic viability during storage. Briefly, the encapsulated probiotic cells were counted by suspending 1 g of sample in 9 mL of PBS, pH 7.2 and concentration at 10^9^ CFU/mL dropped on MRS agar with CaCO_3_ and sub -cultured into MRS broth. Each strain was adjusted to 10^9^ CFU/mL with MRS broth and spotted on MRS agar supplemented with 0.5% (w/v) TDCA and 0.37 g/L of calcium chloride (CaCl_2_) followed by an anaerobic incubation at 37 °C for 72 h. After incubation, the precipitated zone of bile salt (deconjugated form) appeared around the colony was interpreted as a positive result. BSH—producing *Enterococcus faecium*, non-BSH-producing *Lactobacillus* strain and *Escherichia coli* were used as positive control and negative control, respectively.

### Statistical analysis

All experiments were performed in duplicate and repeated at least three times. The results were expressed as means ± standard deviation (n = 5) by using GraphPad program version 5.0 (San Diego, CA, USA). The Tukey’s multiple comparison with one-way ANOVA analysis were used to analyze the variance to compare the differences among various groups. A value of *p* < 0.05 was considered to be statistically significant.

## Results and discussion

### Characteristics of probiotics

BSH activities were observed in *Lactobacillus gasseri* TM1, *Lacticaseibacillus rhamnosus* TM7 (formerly name *Lactobacillus rhamnosus*), and *L. rhamnosus* TM14 isolates. BSH activities of these strains were confirmed in this study and presented in Table 2.

The differences in BSH activities of LAB isolates might be due to the influences of disparate environments on expressions of BSH genes (Song et al., 2019). As an enzyme, BSH recognizes taurine, glycine, or both groups to hydrolyze amide bond between steroid nucleus and amino acid residues of conjugated bile salts. (Ridlon et al., 2006). It converts conjugated bile salts into deconjugated bile salts leading to impairments of lipid emulsification and absorption in the intestine. Moreover, de novo biosynthesis of bile salts in the liver, which is stimulated by bile salt biodegradation, can eliminate stored cholesterol (Song et al., 2019).

### Effect of vegetable powders on probiotic growth

**Fig. 1:**
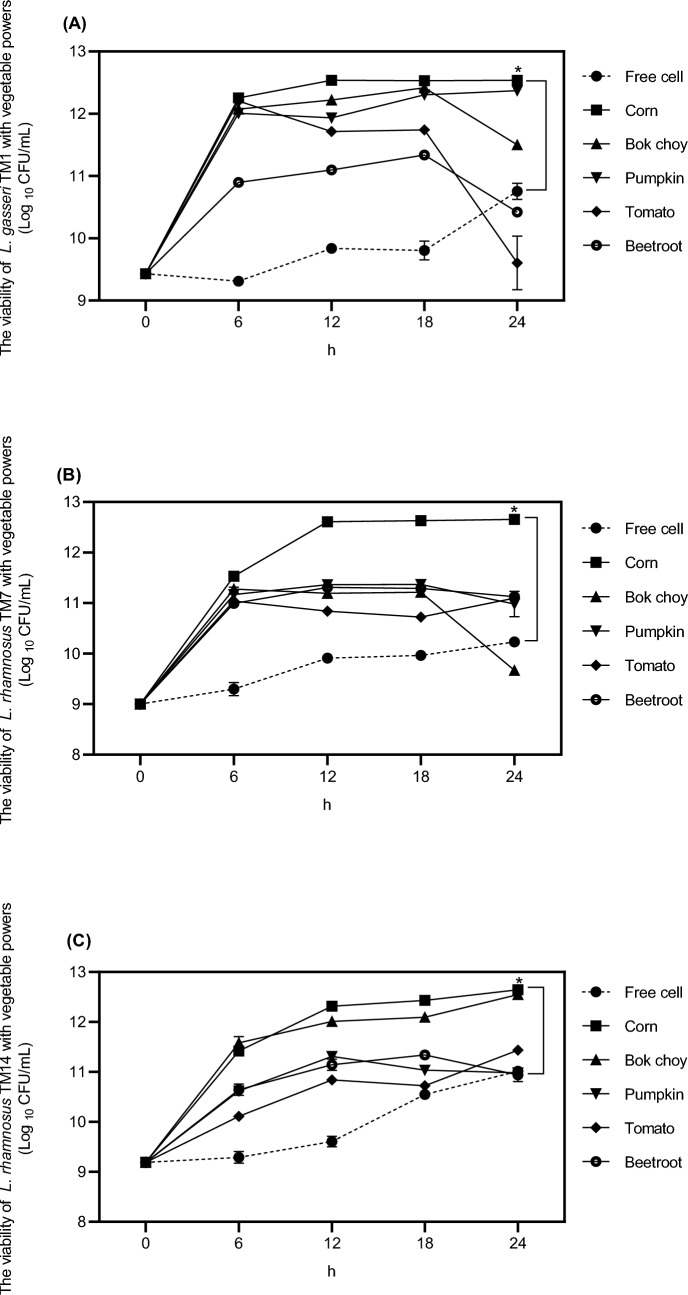
The viability of probiotics *L. gasseri* TM1 (**A**), *L. rhamnosu*s TM7 (**B**), *L. rhamnosus* TM14 (**C**) in different vegetable powers: corn, bok choy, pumpkin, tomato, and beetroot. The data are presented as means ± SD (n = 5). *p < 0.05 compared with free cell

Different vegetable powders including cherry tomato, beetroot, pumpkin, bok choy, and corn at 5% (w/v) were added into culture media of *L. gasseri* TM1, *L. rhamnosu*s TM7 and *L. rhamnosus* TM14. After 24 h of culture, the highest numbers of probiotic cells were observed when corn powder was added into the culture media with 4.3 × 10^12^, 6.5 × 10^12^, and 6.4 × 10^12^ CFU/mL for *L. gasseri* TM1, *L. rhamnosu*s TM7 and *L. rhamnosus* TM14, respectively (Fig. 1). The positive effect of corn powder on the growth of probiotics might be due to its prebiotic property of SCF. With SCF, corn powder provided nutrients to these probiotic strains leading to significant increases in probiotic survival when compared with other vegetable powders (Fig. 1).

Moreover, addition of vegetable powders reduced probiotic deaths and stabilized the growth rates of probiotics during the stationary phase (Fig. 1). Thus, it is suggested that vegetable powders can promote probiotic growths. Probiotics use oligosaccharides, which are biological agents commonly found in vegetables and fruits, as carbon sources in the process of lactic acid fermentation, and produce short chain fatty acids, such as acetic acid, propionic acid and butyric acid. These make pH in the intestines unsuitable for the growth of pathogens as well as stimulate the absorption of calcium and boost the immune system (Delcour et al., 2016). Moreover, xylooligosaccharides (XOS) can be found in corn powder (Samanta et al., 2012), and fructooligosaccharides (FOS), which is found mainly in cherry tomato, beetroot, pumpkin, and bok choy, can withstand acidic conditions and enzymes in the digestive system due to their stable structures. Previous study showed that fermentation of probiotics with vegetable juice can enhance probiotic growths. The number of *L. casei* 431 was enhanced from 10^7^ to 10^8^ CFU/mL after a 6 h of fermentation with beetroot juice (Gamage et al., 2016). In addition, fortification of tomato powder in yogurt containing *L. paracasei* F19 reduced firmness and viscosity of the yogurt, as well as slower pH reduction when comparted to a control yogurt (Demirci et al., 2020).

This study was the first study which integrated corn powder into the mixture of microcapsule material. Several studies have reported prebiotic effects of corn compositions. Consumption of SCF for two weeks significantly enhanced bifidobacterial numbers in healthy volunteers (Costabile et al., 2016). In addition, significant increases in fecal *Lactobacillus* spp. and *Bifidobacterium* spp. counts were reported in healthy subjected received XOS-enriched rice porridge daily for 6 weeks, compared to that of placebo rice porridge (Lin et al., 2016). While corn powder itself may not be a significant source of XOS, it contains resistant starch and dietary fiber with prebiotic properties contributing to gut health (Lamsal and Faubion, 2009). Moreover, corn powder is also a good source of dietary fiber including both insoluble and soluble fibers which support digestive health by improving bowel regularity and serving as a substrate for beneficial gut bacteria (Mussatto and Mancilha, 2007). Therefore, addition of corn powder in the mixture would benefit probiotics by providing more nutrients leading to growth enhancement that can maintain the quantity of probiotics during storage at more than 10^6^ CFU/mL as required by FDA (Colombo et al., 2018).

### Microencapsulation yields before–after freeze drying (probiotic microencapsulated yields)

**Fig. 2:**
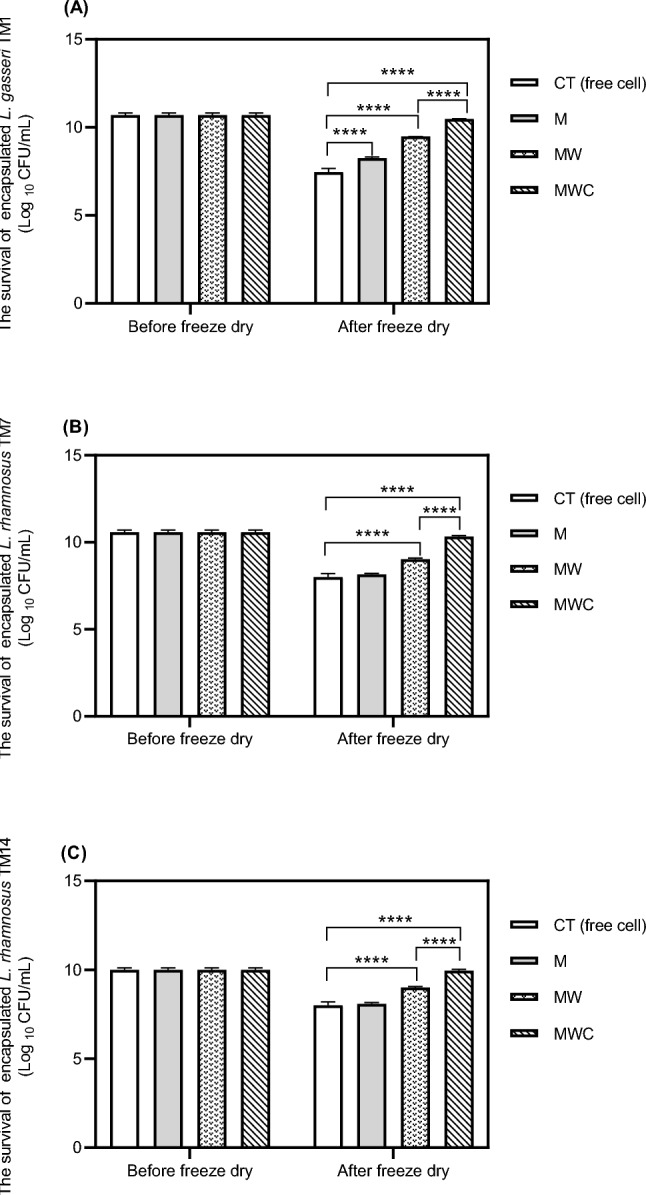
The survival of probiotic free cells and encapsulated of probiotics before–after freeze drying. *L. gasseri* TM1(**A**), *L. rhamnosu*s TM7 (**B**), *L. rhamnosus* TM14 (**C**). Data are represented as means ± SD (n = 5). ****p < 0.0001 compared with free cell. *M* number of probiotics encapsulated with maltodextrin, *MW* number of probiotics encapsulated with maltodextrin and whey protein, *MWC* number of probiotics encapsulated with maltodextrin, whey protein, and corn powder, *CT* free cells used as a control

Before freeze drying, there were approximately 10^10^ CFU/mL of probiotics in each formulation. At the end of the microencapsulation process (after freeze drying), the number of probiotic cells in each microcapsule formulation significantly differed. As expected, the least numbers of probiotics were observed when they were in the form of free cell (CT) (Fig. 2). In formula M and MW, probiotic survivals were decreased after freeze drying process. However, the highest numbers of probiotics (between 2.4–3.0 × 10^10^ CFU/mL) were observed when MWC was used as a microencapsulation material, and this material significantly enhanced viabilities of *L. gasseri* TM1, *L. rhamnosus* TM7 and *L. rhamnosus* TM14 by approximately 29%, 23% and 20%, respectively, when compared with the control with *p*  <  0.0001 (Fig. 2). Moreover, viability of probiotics encapsulated with MWC was also significantly higher than that of MW (*p * <  0.0001). In addition, the numbers of these probiotics before and after freeze drying were comparable suggesting that the mixture of WPI-MD and corn can maintain survival of probiotics during freeze drying process. A strong encapsulated complex can be formed in the mixture of MW and MWC through Maillard reaction. This complex can protect probiotics from extremely low temperatures during the production process. The highest probiotics survivals of these three strains were observed when corn powder was added into the mixture of MW (Fig. 2). Fibers in corn powder form denser and thicker coating thus providing better protection to probiotics (Misra et al., 2022). Previous research reported that corn contains xylooligosaccharides (XOS), a natural component of corn composed of xylose sugar connected by glycosidic bonds at β (1–4) with degrees of polymerization (DP) of 2 to 10 molecules (Ipar et al., 2015). This structure favors microorganisms in which they can reside and protect themselves (Fei et al., 2020). Therefore, the incorporation of corn powder into microcapsules could maintain probiotic viability.

WPI and MD have been wildly used as a mixture for microencapsulation because of their Maillard reaction’s property. Corn which is often used as a raw material in many food industries due to its low cost has never been used as encapsulate material. Structure of corn powder is composed of carbohydrates, soluble corn fiber and fibers which are suitable to be used as a wall material. Thus, incorporation of corn powder into the mixture of WPI-MD might strengthen encapsulated surface. It has been reported that chemically modified whey protein and cornstarch formulated into resistant starch (RS) used as an encapsulated material exhibited significantly greater microencapsulation property and protection against environmental microbial destruction than RS alone (*p*  <  0.05) (Chen et al., 2018).

### Morphological analysis of microcapsule by scanning electron microscope

**Fig. 3:**
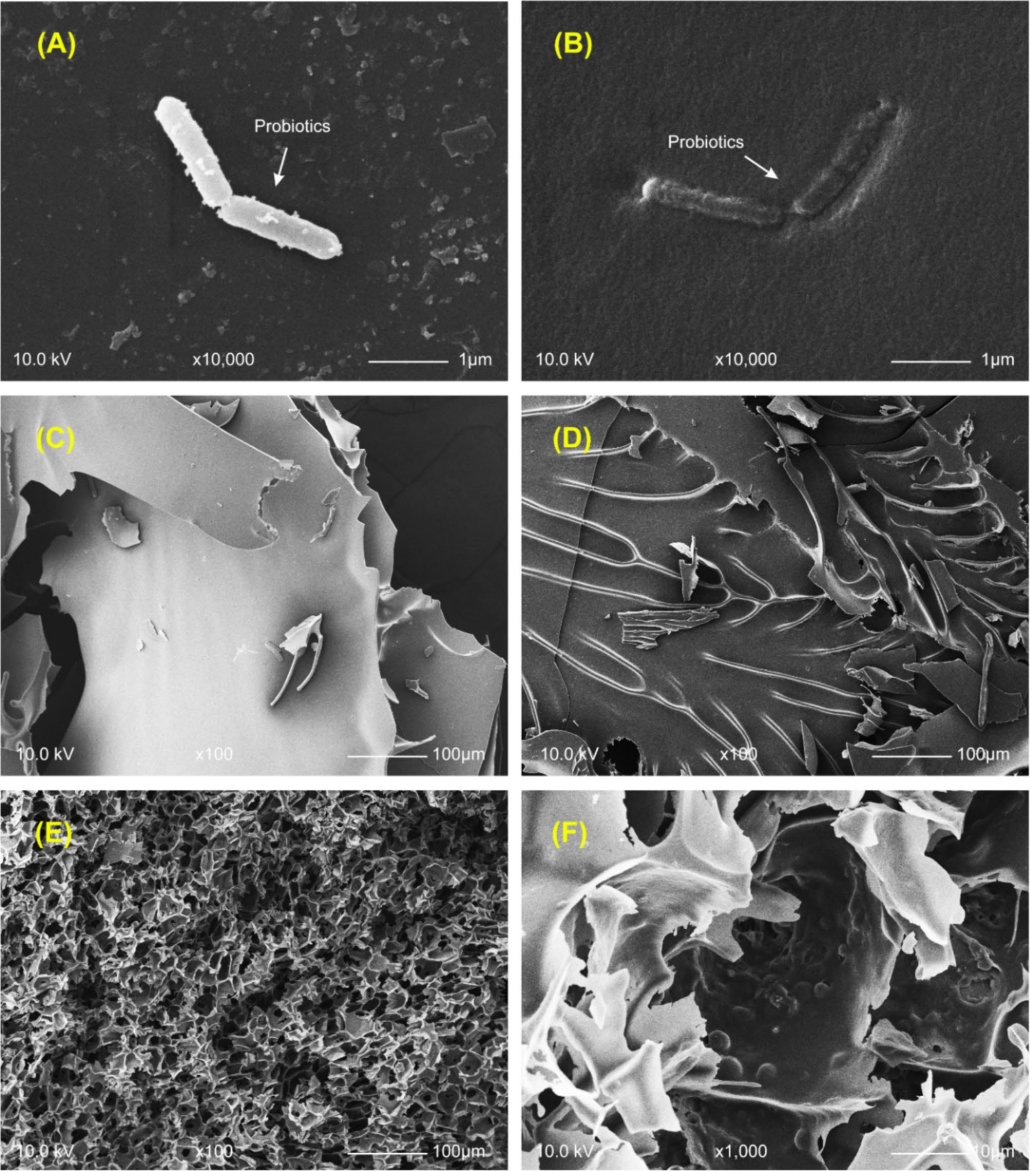
The morphology of probiotics microencapsulated. Scanning electron microscopy (SEM) of free cells (**A**) and encapsulated probiotics (**B**) at 10.0kV with magnification × 10,000. The surface of formula probiotics encapsulated with maltodextrin; MD (**C**), probiotics encapsulated with maltodextrin and whey protein; MW (**D**), and probiotics encapsulated with maltodextrin, whey protein, and corn powder; MWC (**E**). at 10.0kV with magnification × 100. And The surface of formula 3 were zoom-in at 10.0kV with magnification × 1000 (**F**)

Free cells and microencapsulated probiotics were observed under a scanning electron microscopy (SEM) (Fig. 3A, B) The results showed that microencapsulation successfully encapsulated probiotics. Scanning electron microscope was used to characterize the morphology of microencapsulated probiotics which indicates an effectiveness of microencapsulation by freeze drying method. As shown in Fig. 3C, D, E, the surface of microcapsule when MD was used as a material was smooth without burrs. More complex structures were observed when MW and MWC were used as wall materials. Microcapsules of formula MW showed folded sheet structures with smooth surfaces (Fig. 3D), whereas microcapsules of formula MWC had irregular jagged surfaces with dense outer structure (Fig. 3E).

It was the first time that this structure was studied and reported. Combination of WPI, MD, and corn formed a reticulum-like structure wrapped in another layer which potentially increasing the encapsulation efficiency (Yan et al., 2020). The corn powder used in this study was provided by Bio Veggie Company and was composed of the entire corn including corn cob, husk and kernels. Corn powder contains fiber components, thus it can be hypothesized that the surface of MWC, as seen in the Fig. 3E, and F, is fiber that helps to form a denser wall material than formula MW for encapsulating probiotics (Shariff et al., 2016). The alteration in the surface of the encapsulating material may affect the survival of probiotics due to the presence of fiber, which helps create a denser encapsulating structure compared to formula MW. Additionally, the soluble fiber from corn, which encapsulates the probiotics, is not digested in the stomach, allowing the wall material to better withstand the acidic conditions in the stomach (Jenkins et al., 2021). This type of fiber can dissolve in water and form a gel when dissolved. The breakdown of soluble fiber typically occurs in the large intestine (Slavin, 2013). These properties enhance the survival of probiotics in the acidic environment of the stomach, making this formula more effective in delivering probiotics to the target organs compared to formula MW.

### Survival of microencapsulated probiotics in simulated gastrointestinal conditions

Survivals of microencapsulated probiotics in simulated gastrointestinal conditions were examined using artificial gastric and intestinal fluids. The numbers probiotics free cells were decreased from an initial number of 10^9^ CFU/mL to approximately 10^8^–10^7^ CFU/mL after 3 h incubation in artificial gastric fluid (Table 1). However, no live probiotics survived acidic pH (pH 2) of artificial intestinal fluid at 3 h (Table 1). At this strong acidic solution, cell walls of probiotics will break down leading to the death of probiotics. It has been demonstrated that probiotics need to go through microencapsulation processes before being used in an industry or as dietary supplements (Abbas et al., 2023). The reductions of probiotic viabilities from an initial number of 10^8^ CFU/mL to roughly 10^5^ CFU/mL in artificial gastric and intestinal fluids were observed when MD alone was used as microencapsulated material (Table 1) suggesting that this material may not provide sufficient protection to probiotics once inside the body. On the other hand, the numbers of probiotics in both artificial GI fluids were increased to approximately 10^10^ CFU/mL from the starting numbers when MW and MWC were used (Table 1). Encapsulation of probiotics with either MW or MWC can protect probiotics from destruction caused by various environment and allow probiotics to survive better in these conditions. Moreover, MW or MWC can also act as a delivery vehicle for probiotics to their target organs, the small intestine, allowing more probiotics to bind to that area and provide full benefits to the body. Probiotics will be protected and maintained from GI juices by these capsules, which are the conjugation between amino acid and short-chain carbohydrates (Rodrigues et al., 2020). This study is in consistent with previous study reported that addition of prebiotics including inulin, rice bran or hi-maze into pectin which was a main active ingredient of microencapsulation enhanced survival of *L. acidophilus* in the gastrointestinal simulation and maintained probiotics viability for longer storage period than pectin alone (Raddatz et al., 2019).

Moreover, Table 1 does not merely present the survivability of probiotics but also demonstrates the efficiency of MWC in encapsulating probiotics. Even though the survival rates of microencapsulated probiotics followed the same trend, the number of probiotics microencapsulated with MWC in the artificial intestinal fluid reached up to 10^9^ CFU/mL after 6 h incubation. This indicates the encapsulation efficiency of MWC which allows the probiotics to reach the target organ most effectively when compared to other groups. Conversely, the probiotics in the MW group showed the highest numbers in artificial gastric fluids, meaning that the probiotics are secreted from the encapsulating material in artificial gastric fluids conditions, which is not the target organ for probiotics. This could lead to probiotics being destroyed by stomach acid. Therefore, it can be concluded that enhancing the encapsulation efficiency by adding corn powder can protect the probiotics, making them more resistant to artificial gastric fluids conditions.

**Table 1:** The survival of probiotic free cells and encapsulated *L. gasseri* TM1 under simulated gastric juice and simulated intestinal juice conditions

Formula	Number of viability (Log CFU/mL)						
	Initial	Artificial gastric fluids			Artificial intestinal fluids		
		1 h	2 h	3 h	4 h	5 h	6 h
*L. gasseri *TM1							
CT	9.361 ± 0.05^aA^	9.041 ± 0.190^aA^	8.602 ± 0.102^aA^	8.00 ± 0.010^aA^	0	0	0
M	8.014 ± 0.104^aA^	7.512 ± 0.102^bB^	7.115 ± 0.15^cB^	6.815 ± 0.105^cB^	6.360 ± 0.110^eB^	6.12 ± 0.107^eB^	6.00 ± 0.05^eB^
MW	8.653 ± 0.15^aA^	8.929 ± 0.105^aA^	10.439 ± 0.41^dA^	10.243 ± 0.10^dA^	9.929 ± 0.04^bA^	9.778 ± 0.05^bA^	8.301 ± 0.05^aA^
MWC	7.30 ± 0.15^aA^	7.525 ± 0.160^aA^	8.452 ± 0.41^dA^	8.650 ± 0.10^dA^	10.254 ± 0.04^bA^	10.00 ± 0.05^bA^	9.58 ± 0.05^aA^
*L. rhamnosus *TM7							
CT	9.243 ± 0.05^aA^	8.653 ± 0.190^aA^	8.602 ± 0.102^aA^	8.00 ± 0.010^aA^	0	0	0
M	8.519 ± 0.108^aA^	7.301 ± 0.010^bB^	7.00 ± 0.072^cB^	6.284 ± 0.115^dB^	6.01 ± 0.320^eB^	5.367 ± 0.048^eB^	5.106 ± 0.005^eB^
MW	9.452 ± 0.35^aA^	10.20 ± 0.78^aA^	10.447 ± 0.10^dA^	10.778 ± 0.25^cA^	9.301 ± 0.105^aA^	9.01 ± 0.048^aA^	8.512 ± 0.110^aA^
MWC	6.550 ± 0.305^aA^	7.00 ± 0.170^aA^	7.52 ± 0.105^dA^	7.98 ± 0.25^cA^	10.00 ± 0.455^aA^	9.820 ± 0.408^aA^	9.05 ± 0.20^aA^
*L. rhamnosus *TM14							
CT	9.278 ± 0.067^aA^	8.954 ± 0.102^aA^	8.176 ± 0.30^aA^	7.698 ± 0.145^aA^	0	0	0
M	8.140 ± 0.600^aA^	7.845 ± 0.125^bB^	7.501 ± 0.01^cB^	6.450 ± 0.230^dB^	6.205 ± 0.110^eB^	6.07 ± 0.405^eB^	5.95 ± 0.085^eB^
MW	9.20 ± 0.108^aA^	9.929 ± 0.108^aA^	10.74 ± 0.035^aA^	10.45 ± 0.065^aA^	10.04 ± 0.505^aA^	9.653 ± 0.140^aA^	9.107 ± 0.750^aA^
MWC	6.507 ± 0.108^aA^	7.000 ± 0.108^aA^	7.250 ± 0.035^aA^	7.860 ± 0.065^aA^	10.658 ± 0.505^aA^	10.754 ± 0.140^aA^	10.25 ± 0.750^aA^

Data are represented as means ± SD (n = 5)

*A-B* Significantly different (*P* < 0.05) in the same column, *a-e* Significantly different (*P* < 0.05) in the same row, *M* MD alone, MW; the mixture of MD-WPI, *MWC* the mixture of MD-WPI and corm powder, *CT* free cells were used as a control

### Survival of microencapsulated probiotics and free cells during long-term storage

**Fig. 4:**
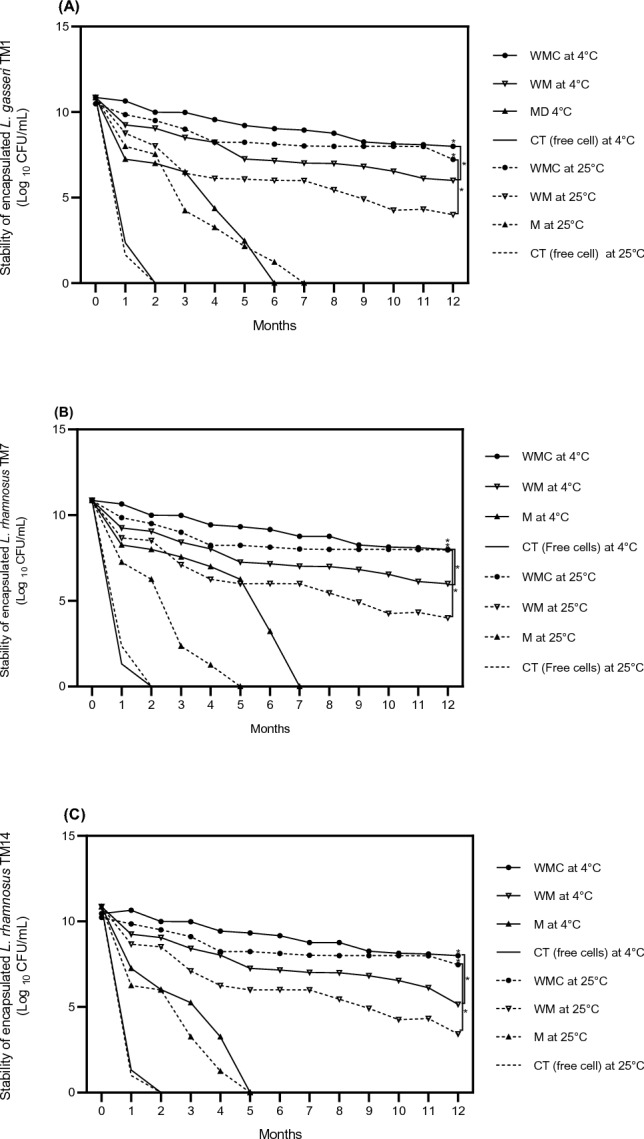
Stability of probiotics encapsulated and free cells during long-term storage at 4 °C and 25 °C. M was MD alone. MW was the mixture of MD-WPI. MWC was the mixture of MD-WPI, and CT was free cells used as a control. Data are represented as means ± SD means (n = 5). ****p > 0.001 compared with free cell

Viabilities of free cells (CT) and microencapsulated probiotics stored at 4 °C and 25 °C (room temperature) for 1 year are shown in Fig. 4. After 12 months of storage at 4 °C and 25 °C, the viability of three probiotic strains (10^11^ CFU/mL) encapsulated in MWC significantly reduced to approximately 2.48 × 10^8^–10^9^ CFU/mL (Fig. 4). The numbers of live probiotics were much decreased when other mixtures were used as wall materials. The numbers of live probiotics were significantly decreased to roughly 7.56 × 10^4^–10^6^ CFU/mL and 4.41 × 10^2^–10^3^ CFU/mL when microencapsulated with MW and M, respectively (Fig. 4). Moreover, in the form of free cell (CT), these strains of probiotics could survive in the storage environment for only 2 months due to lacking of protective material and sufficient nutrients (Maia et al., 2023). The formulation WPI and MD used in combination with the freeze-drying process could lead to an enhancement of probiotic survival rate (Khosravi Zanjani et al., 2018). Previous studies reported the improvement of microencapsulated probiotic cell survival during storage. Microencapsulation of *L. plantarum* CCMA 0359 by spray drying using whey powder as the wall material enhanced probiotic viability during 7 °C storage for 90 days when incorporated into cream cheese (Andrade et al., 2023). Microencapsulate probiotics in a vegan product were able to resist acidic pH (pH 2), temperature at 45 °C, and storage conditions (Raddatz et al., 2022). In this study, all 3 strains of probiotics were well protected during storage at 4 °C and 25 °C when the mixture of MWC was used as the wall material. The numbers of survival probiotics in these groups meet the criteria by the FDA which requires a minimum of 10^6^ CFU/mL of probiotics in products throughout storage (Colombo et al., 2018). Our findings agree with a previous study which suggests that the wall materials should contain more than two types of ingredients to improve encapsulation efficiency above the formula with one or two types of ingredients (Anchalee and Ubonrat, 2020).

### Confirmation of bile salt hydrolase enzyme activity of probiotics under each experimental condition

Bile salt hydrolase enzyme activities were maintained in microencapsulated probiotics. These probiotics continued to produce BSH with the same potency as probiotics without microencapsulation as shown in Table 2. In addition, the stability of BSH production by microencapsulated probiotics was preserved as shown by the precipitation zone (Supplementary result). The result of the BSH enzyme activity demonstrates that after the freeze-drying process, the probiotics are still able to secrete the BSH enzyme. This result demonstrated that the test mixtures used for microencapsulation can protect and maintain probiotics viability from the freeze-drying process, simulated gastrointestinal conditions, and storage conditions (4 °C and 25 °C). Thus, the mixture of MWC is beneficial to probiotics and can be further developed at the industrial level.

**Table 2:** Bile salt hydrolase (BSH) enzyme activity of probiotics under each experimental condition

Condition		BSH activity											
		*L. gasseri* TM1				*L. rhamnosus* TM7				*L. rhamnosus* TM14			
		CT	M	MW	MWC	CT	M	MW	MWC	CT	M	MW	MWC
Before freeze drying		+ + +	+ + +	+ + +	+ + +	+ + +	+ + +	+ + +	+ + +	+ + +	+ + +	+ + +	+ + +
After freeze drying		+ + +	+ + +	+ + +	+ + +	+ + +	+ + +	+ + +	+ + +	+ + +	+ + +	+ + +	+ + +
Artificial gastric fluids	0 h	+ + +	+ + +	+ + +	+ + +	+ + +	+ + +	+ + +	+ + +	+ + +	+ + +	+ + +	+ + +
	1 h	+ +	+ + +	+ + +	+ + +	+ +	+ + +	+ + +	+ + +	+ + +	+ + +	+ + +	+ + +
	2 h	+ +	+ + +	+ + +	+ + +	+ +	+ + +	+ + +	+ + +	+ + +	+ + +	+ + +	+ + +
	3 h	+ +	+ + +	+ + +	+ + +	+ +	+ + +	+ + +	+ + +	+ + +	+ + +	+ + +	+ + +
Artificial intestinal fluids	4 h	−	+ + +	+ + +	+ + +	−	+ + +	+ + +	+ + +	−	+ + +	+ + +	+ + +
	5 h	−	+ + +	+ + +	+ + +	−	+ + +	+ + +	+ + +	−	+ + +	+ + +	+ + +
	6 h	−	+ + +	+ + +	+ + +	−	+ + +	+ + +	+ + +	−	+ + +	+ + +	+ + +

Since the BSH activity table includes the test results of BSH after freeze-drying and testing in a simulated gastrointestinal condition compared to before freeze-drying, it shows that the freeze-drying process does not destroy the probiotic activity

The bile salt hydrolase (BSH) was measured according to the diameter of the colony precipitation around TDCA agar (n = 3, mean ± SD): -Probiotics do not grow, +  + ; ≤ 8.0 mm, +  +  + ; ≤ 8.1–13.0 mm. M was MD alone, MW was the mixture of MD-WPI, MWC the mixture of MD-WPI, and CT was free cells used as a control

## Supplementary Information

Below is the link to the electronic supplementary material.

Supplementary file1 (DOCX 240 kb)
