# Supplementary material for: Freeze drying microencapsulation using whey protein, maltodextrin and corn powder improved survivability of probiotics during storage
Source: Food Sci Biotechnol. 2024 Sep 12;34(4):959–70. doi: 10.1007/s10068-024-01706-w (PMC11832850; doi:10.1007/s10068-024-01706-w)
Supplement: Supplementary file 1 — Supplementary file1 (DOCX 240 kb) [file 10068_2024_1706_MOESM1_ESM.docx]

**Appendix**

**Supplementary result**


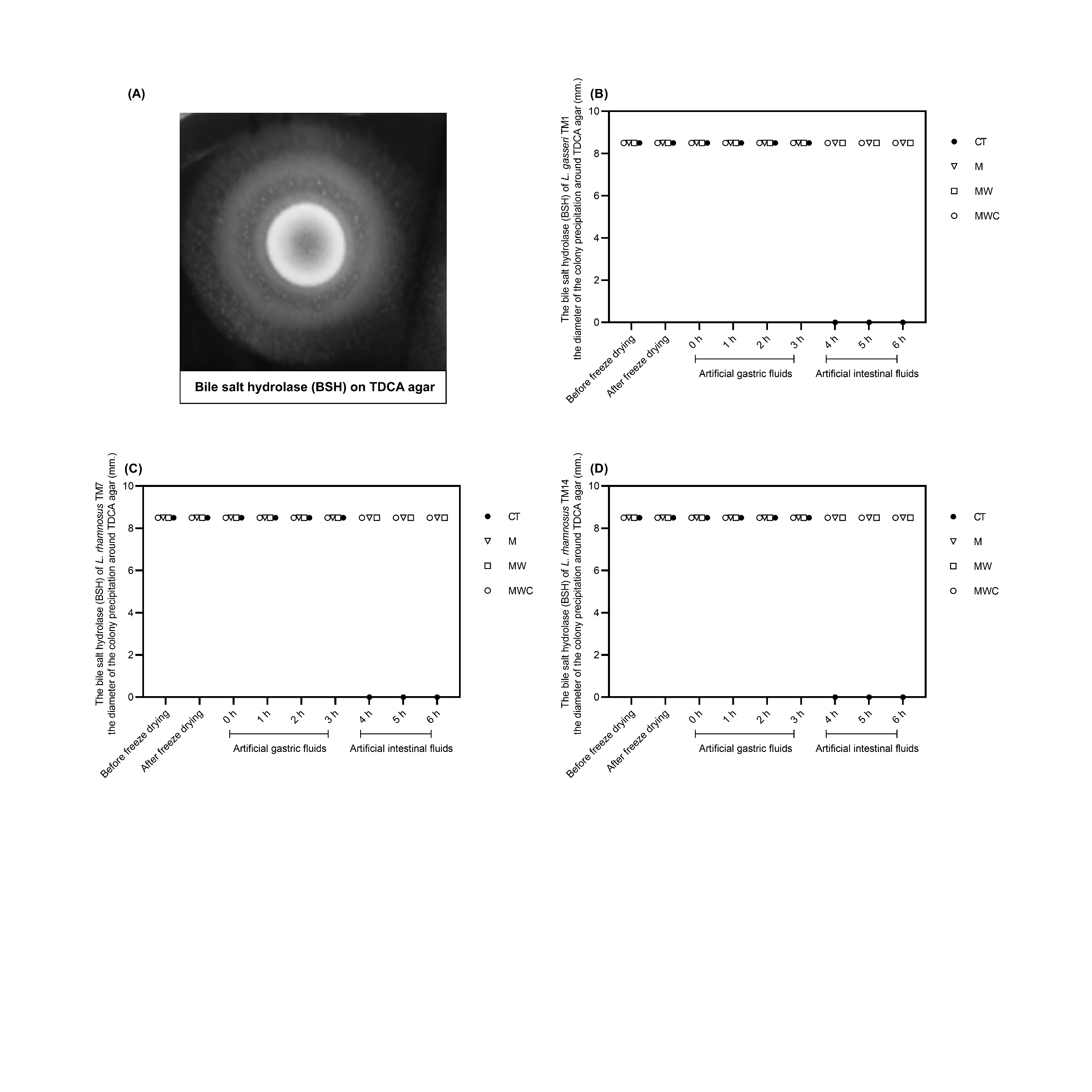


**Supplementary Fig 1.** The bile salt hydrolase (BSH) of probiotics on TDCA agar (A). The bile salt hydrolase (BSH) was measured according to the diameter of the colony precipitation around TDCA agar (n = 3, mean ± SD) (B). M; Formula 1 was MD alone, MW; Formula 2 was the mixture of MD-WPI, MWC; Formula 3 was the mixture of MD-WPI and corm powder, and CT; Free cells were used as a control.
